# Remote enrollment into a telehealth-delivering patient portal: Barriers faced in an urban population during the COVID-19 pandemic

**DOI:** 10.1007/s12553-021-00614-x

**Published:** 2021-11-08

**Authors:** Jordan A. Francke, Phillip Groden, Christopher Ferrer, Dennis Bienstock, Danielle L. Tepper, Tania P. Chen, Charles Sanky, Tristan R. Grogan, Matthew A. Weissman

**Affiliations:** 1 University of California, 757 Westwood Plaza, Los Angeles, CA 90095 USA; 2 Icahn School of Medicine, Mount Sinai, New York, NY 10029 USA; 3 Mount Sinai Downtown, Department of Medicine, New York, NY 10003 USA; 4 New York Eye and Ear Infirmary, Department of Medicine, New York, NY 10003 USA; 5 Department of Statistics, University of California, Los Angeles, CA 90095 USA

**Keywords:** Telemedicine, Barriers to Care, COVID-19

## Abstract

Telehealth drastically reduces the time burden of appointments and increases access to care for homebound patients. During the COVID-19 pandemic, many outpatient practices closed, requiring an expansion of telemedicine capabilities. However, a significant number of patients remain unconnected to telehealth-capable patient portals. Currently, no literature exists on the success of and barriers to *remote* enrollment in telehealth patient portals. From March 26 to May 8, 2020, a total of 324 patients were discharged from Mount Sinai Beth Israel (MSBI), a teaching hospital in New York City. Study volunteers attempted to contact and enroll patients in the MyChart patient portal to allow the completion of a post-discharge video visit. If patients were unable to enroll, barriers were documented and coded for themes. Of the 324 patients discharged from MSBI during the study period, 277 (85%) were not yet enrolled in MyChart. Volunteers successfully contacted 136 patients (49% of those eligible), and 39 (14%) were successfully enrolled. Inability to contact patients was the most significant barrier. For those successfully contacted but not enrolled, the most frequent barrier was becoming lost to follow-up (29% of those contacted), followed by lack of interest in remote appointments (21%) and patient technological limitations (9%). Male patients, and those aged 40–59, were significantly less likely to successfully enroll compared to other patients. Telehealth is critical for healthcare delivery. Remote enrollment in a telemedicine-capable patient portal is feasible, yet underperforms compared to reported in-person enrollment rates. Health systems can improve telehealth infrastructure by incorporating patient portal enrollment into in-person workflows, educating on the importance of telehealth, and devising workarounds for technological barriers.

## Introduction

Telemedicine has shown the potential to add tremendous value to the healthcare field. Over the last decade, numerous health systems have increased their investment in this sector;from 2010 to 2017, the number of hospitals with a fully or partially implemented telemedicine program more than doubled from 35 to 76% [1]. A prominent benefit of telehealth is the efficiency of care for both patients and providers. Including time spent in-transit and within the waiting area, one study found that the average American spends over 120 min at a doctor’s appointment [2]. Thus, to save time, patients have shown interest in completing telehealth visits even when in-person appointments were available [2]. Telehealth services also provide a means of increasing access to care— allowing providers to reach the nearly 400,000 patients in the US who are completely, and the 1.5 million patients who are mostly, homebound [3, 4]. In recent years, telehealth has expanded beyond the confines of primary care [5], and has been evolved into delivering more specialized services effectively using a combination of video and phone appointments, including pathology [6], oncologic care and chemo supervision [7], psychiatry and other mental health services [8], addiction counseling and prescriptions for harm reduction (e.g., buprenorphine) [9], physical therapy [10], and emergency medicine care [11]. Some medical education programs have even adopted innovative mechanisms to involve trainees in their telehealth delivery platforms to provide learners with clinical opportunities when there is a shortage of in-person clinic appointments [12]. These adaptations evolved out of a complex, rapidly changing medical world driven by competition where inventive new ideas offering convenience and efficiency are rewarded with increased consumer engagement as well as potential (temporary) profit monopolies [13, 14]. These systems have been described as a sort of technological parasitism, where old technologies are devoured by newer ones providing consumers with quicker and more effective results [15, 16].

Despite its significant advantages, ubiquitous acceptance of telemedicine as a care option has faced significant hurdles. Limited interstate licensure and insurance coverage policies have logistically restricted telehealth’s widespread usage, restricted telehealth delivery to video visits and not telephonic visits, and hindered service and payment parity across even geographically proximal states [17–20]. In addition, one study reported that, on top of fears of technology, remote visits threatened patients’ identity and perceived independence, with many respondents stating that they associate telehealth with high dependency and ill health [21]. However, data do show that the majority of patients and providers believe that the quality of care provided during virtual visits is the same, if not better, compared to in-person consultations [22].

The surge of COVID-19, the disease caused by the novel SARS-CoV-2 virus, in New York City (NYC) was an important inflection point in urban usage of telehealth. A number of risk factors for transmission of COVID-19 have been described, including age (with elderly being highest at risk), male sex, crowded living and/or working environments, and chronic medical illnesses like diabetes mellitus, kidney disease, malignancy and cardiovascular disease predisposing patients to more severe disease [23]. Due to the challenges of providing safe outpatient treatment without crowding, many practices limited in-person visits and turned preferentially to telehealth to provide ongoing care— transforming it from a useful tool to a patient care necessity. It is estimated that in the first quarter of 2020, telehealth visits (for COVID-related and -unrelated conditions) increased by 50% compared to the prior quarter, with a 154% surge in the last week of March 2020 compared to the same week in 2019 [24]. This trend continued throughout the year and by the end of 2020, the Centers for Disease Control and Prevention reported in-person ambulatory care (i.e., outpatient) medical visits had plummeted by as much as 60%, and telehealth appointments had increased by 30% [25].

Our study was interested in exploring the potential for remote enrollment in a telehealth-delivering patient portal in an urban setting. Rural populations have been well described as being difficult to engage in telemedicine due to difficulty obtaining reliable internet, computer or smart phone technologies needed to access healthcare portals, or even education that telehealth exists [26]. Conversely, health systems have also had unique struggles recruiting urban populations to engage in telehealth platforms, due to socioeconomic challenges as well as healthcare markets already being saturated with an abundance of in-person providers [27]. While success rates for in-person telehealth portal enrollment among some urban patient populations have been reported to be as high as 87% [28], minimal published data have addressed enrollment rates when conducted remotely (i.e., electronically by means of either telephone or e-mail communication) [29]. Given the possibility for future limitations of in-person activities due to surges of new variants of COVID-19 (including the delta variant) or other infectious pathogens [30–32], we sought to understand the success of and barriers to remotely enrolling patients in an online patient portal and assess whether this presents a feasible route for scaling telemedical infrastructure. The study had three key research questions:(1) is remote enrollment in a telehealth portal a viable alternative to in-person enrollment, (2) do certain vulnerable subpopulations have statistically significant differences in enrollment success, and (3) what are the barriers to enrollment for those who do not enroll? We hypothesized that remote enrollment would have equal success, if not higher success, compared to in-person enrollment due to its convenience and perceived time savings, but that more vulnerable demographic populations (e.g., women, ethnic minorities, the elderly, and non-English speaking patients) would be less likely to enroll in telehealth due to less access to necessary technologies, technological and medical literacy, and trust of healthcare professionals. We hypothesized the key barriers to enrollment would be difficulty contacting patients, and patients discomfort with technology. To our knowledge, this is the first paper to describe patient barriers to remote enrollment in a telehealth portal.

## Materials and methods

### Sample and data

This manuscript represents a retrospective cohort study conducted at Mount Sinai Beth Israel— a teaching hospital located on the Lower East Side of Manhattan, NYC providing care to a culturally diverse and medically underserved patient population. From the period of March 26 to May 8, 2020, over 300 patients were discharged from the inpatient medicine floors for conditions both related and unrelated to infection with SARS-CoV-2, and were recommended to complete a post-discharge telehealth follow-up. May 8th was chosen as the end date as the clinics began to open for both in-person and virtual visits after that date. Mount Sinai Union Square, where discharged MSBI patients receive outpatient follow-up care, uses Epic for its electronic health record (EHR) system (Epic Care;Epic Systems, Verona, Wisconsin). Through this EHR, patients are eligible for MyChart— a patient portal that displays test results, facilitates patient-provider communication, and allows for video-based synchronous (i.e., two-way audiovisual connection) telemedicine delivery through a free mobile application (Epic Care and MyChart;Epic Systems, Verona, Wisconsin).

The MSBI Department of Medicine partnered with a rotating team of up to six student volunteers from the Icahn School of Medicine at Mount Sinai to remotely (i.e., telephonically) enroll individuals in MyChart. The inpatient case managers compiled spreadsheets of patients anticipated to be discharged, which were shared with volunteers daily and contained the following information:patient name, medical record number, date of discharge, and hospital location.

### Measures of variables

The student volunteers attempted to contact patients on both their personal mobile devices, if provided in the EHR, or their hospital rooms’ landline phones. Volunteers were provided with an illustrated flowchart with step-by-step instructions on how to facilitate patient enrollment in MyChart, if they were interested, as well as a document of frequently asked questions when enrolling patients. For patients successfully reached, an email address would be confirmed and an activation link would be sent to that address where the patient or their healthcare proxy could complete the activation while the volunteer remained on the phone to guide them through any questions they faced while registering. Once registered, they would be set up with a telehealth appointment by the appropriate clinic for which they had an active referral order. If patients were not interested in enrolling, the verbatim reason would be documented and was later coded into categories (see below in Data analysis procedure section). If the patients were not reached, at least one subsequent attempt was made to contact them the following day. After each patient had been contacted, the following demographic variables were documented for the encounter:the patient age, sex, ethnicity, and primary language used during the encounter. Any non-English speaking patients received instruction facilitated by a trained, third-party interpreter. If a patient had a healthcare proxy complete the enrollment, this was also documented.

### Data analysis procedure

For those unable to be enrolled, patient-specific barriers to enrollment were copied verbatim from the encounter and subsequently coded. Three authors (JF, PG, and CF) independently reviewed the list of reported barriers, and devised their own subcategories to group the reported themes. They then met with the remaining authors and, through comparative analysis, agreed upon the classification system that most effectively and comprehensively organized the uncoded content. For the demographic variables, descriptive statistics were utilized to analyze the cohort characteristics of the study. Associations between demographic factors (e.g., age, ethnicity, primary language) were compared across different aspects of the care cascade (e.g., successful contact made, MyChart enrollment) using the chi-square test. When the overall test was significant, we ran pairwise follow-up tests. We also ran the t-test to compare age between groups, as this was the one continuous demographic variable. Statistical comparisons were computed using IBM SPSS V27 (Armonk, NY) and p-values < 0.05 were considered statistically significant.

## Results

### Patient demographics

During the study period, a total of 324 patients were discharged from the MSBI inpatient medicine floor. Demographic data were analyzed descriptively for this cohort and can be seen in Table 1. To provide a snapshot of the 324 patients in the cohort, approximately 40% (*n* = 132) identified as female, 44% (*n* = 144) were aged 60–89 years old, 23% self-identified their ethnicity within the medical record as “African-American, Afro-Caribbean, Black (Non-Hispanic),” and 84% spoke English as their primary language. The median age for the cohort was 66 (ranging from 21 to 96 years old, with a standard deviation of 15.6 years).

**Table 1:** Cohort Demographic Characteristics

**Characteristic**	**Demographics, *n* (*%*)**
***Gender***	
*Male*	190 (*58%*)
*Female*	132 (*40%*)
*Transgender/Non-binary*	2 (*1%*)
***Age***	
*0–19 years*	0 (*0%*)
*20–39 years*	23 (*7%*)
*40–59 years*	89 (*27%*)
*60–79 years*	144 (*44%*)
*80–99 years*	68 (*21%)*
***Ethnicity/Race***	
*African-American, Afro-Caribbean, Black (Non-Hispanic)*	74 *(23%)*
*Asian/Pacific Islander*	36 *(11%)*
*Hispanic*	57 *(18%)*
*White/Caucasian (Non-Hispanic)*	70 *(22%)*
*Other or Unknown*	87 *(27%)*
***Primary Language***	
*Chinese (Mandarin, Cantonese, or not specified)*	10 *(3%)*
*English*	273 *(84%)*
*Russian*	3 *(1%)*
*Spanish*	30 *(9%)*
*Other*	3 *(1%)*
*Not specified and unable to contact*	5 *(2%)*
***TOTAL***	***N* = *324***

### Contact and enrollment success

**Fig. 1:**
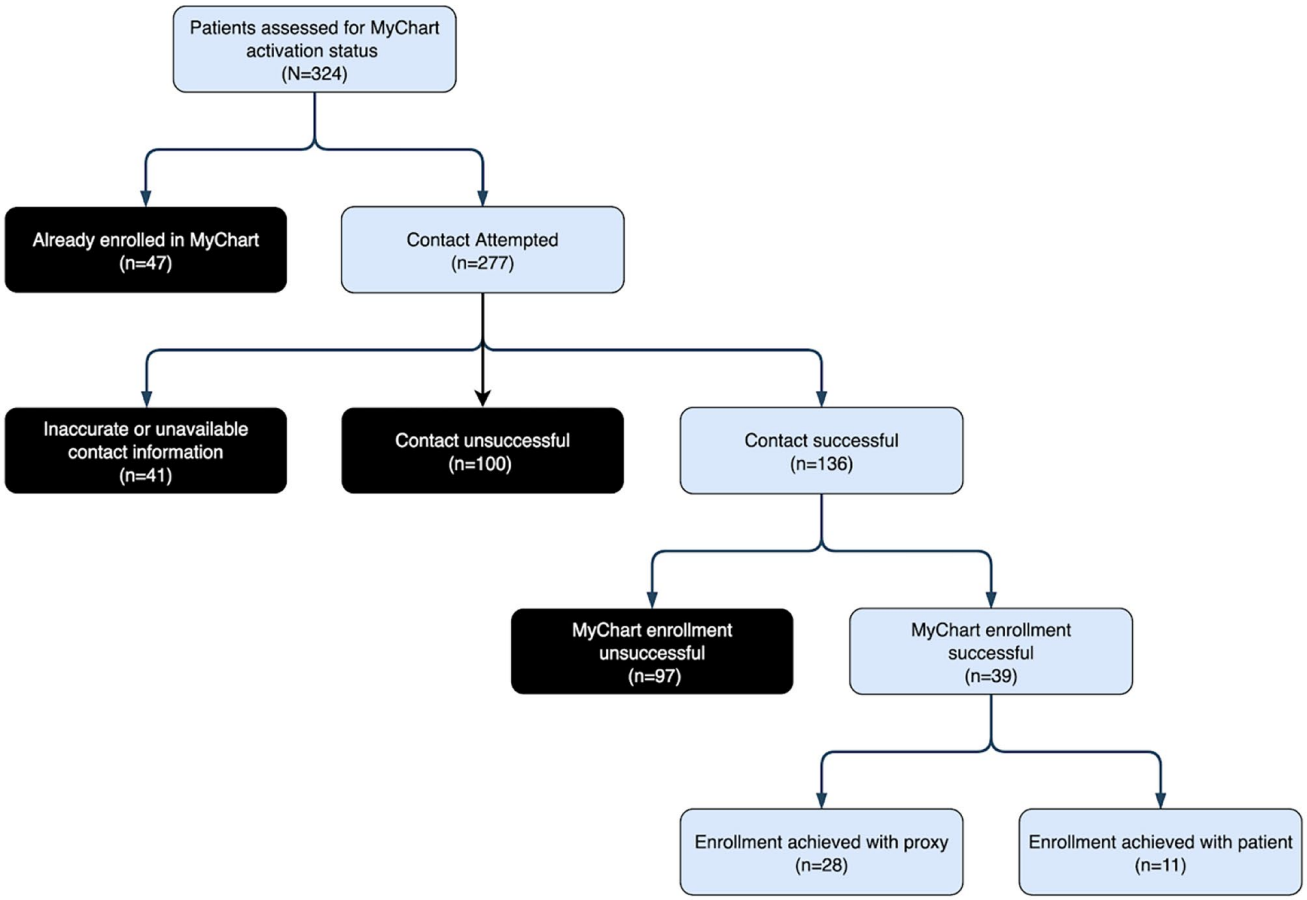
Cascade of patient enrollment:Of the original cohort (*n* = 324), 277 were eligible to be enrolled. A total of 136 patients were successfully contacted, and 39 were successfully enrolled (28 via healthcare proxy)

The cascade of care is shown in Fig. 1. Of the 324 discharged patients, a total of 47 (15%) were already enrolled in MyChart and therefore not contacted. Of the 277 unenrolled patients, 141 (51%) were unable to be reached through either their hospital landline or mobile phones, if the latter was provided within the EHR. A total of 41 patients (15%) had either no personal telephone number provided in their chart, or the number provided was out of service. Of the 136 unenrolled patients who could be contacted, 39 (29% of those contacted) successfully activated their MyChart account. While these individuals represent an 83% increase in total MyChart enrollment within the cohort, just 14% of those eligible to be enrolled actually activated their accounts. Nearly three-quarters of these successful enrollments (72%;*n* = 28) were completed through a healthcare proxy, which included spouses, adult children, or paid caregivers.

Of those successfully enrolled, approximately half were male (*n* = 19, 49%). Nine (23%) self-identified as white, six (15%) identified as African-American, seven (18%) as Asian American, and seven (18%) as Hispanic, and 10 (26%) did not have an identified ethnicity in their chart or it was specified as “Other.” Zero enrolled patients were in the 0–19 age category, one (3%) was 20–39 years old, two (5%) were 40–59 years old, twenty-two (56%) were 60–79 years old, and fourteen (36%) were 80–99 years old.

### Statistical differences in enrollment rates

Statistical differences were investigated along 4 different points of the care cascade in Fig. 1:(1) whether or not patients were already enrolled in MyChart before contact was attempted (Table 2), (2) whether or not patients had missing or inaccurate contact information (Table 3), (3) whether or not patients were successfully contacted (Table 4), and (4) whether or not the contacted patients successfully enrolled in MyChart (Table 5).

**Table 2:** Already Enrolled

	No (*n* = 277)	Yes (*n* = 47)	Chi-square *p* value	Pairwise-follow up test *p* value	T-test* p* value
**Male Sex**	162 (58.9%)	28 (59.6%)	0.932	N/A	N/A
**Age**			**0.037**		0.453
20–39	16 (5.8%)	7 (14.9%)		**0.024**	
40–59	82 (29.6%)	7 (14.9%)		**0.037**	
60–79	120 (43.3%)	24 (51.1%)		0.323	
80–99	59 (21.3%)	9 (19.1%)		0.738	
**Ethnicity**			**0.049**		N/A
African-American, Afro-Carib., Black	66 (23.8%)	8 (17.0%)		0.304	
Asian/Pacific Islander	27 (9.7%)	9 (19.1%)		0.058	
Hispanic	53 (19.1%)	4 (8.5%)		0.077	
White	76 (27.4%)	11 (23.4%)		0.564	
Other/Unknown	55 (19.9%)	15 (31.9%)		0.063	
**English as Primary Language**	231 (84.9%)	42 (89.4%)	0.424	N/A	N/A

**Table 3:** Inaccurate/Missing Contact Information

	No (*n* = 236)	Yes (*n* = 41)	Chi-square *p* value	Pairwise-follow up test *p* value	T-test* p* value
**Male Sex**	135 (57.4%)	27 (67.5%)	0.232	N/A	N/A
**Age**			**0.008**		**0.017**
20–39	12 (5.1%)	4 (9.8%)		0.237	
40–59	62 (26.3%)	20 (48.8%)		**0.004**	
60–79	107 (45.3%)	13 (31.7%)		0.104	
80–99	55 (23.3%)	4 (9.8%)		0.051	
**Ethnicity**			0.996		N/A
African-American, Afro-Carib., Black	57 (24.2%)	9 (22.0%)		N/A	
Asian/Pacific Islander	23 (9.7%)	4 (9.8%)		N/A	
Hispanic	45 (19.1%)	8 (19.5%)		N/A	
White	65 (27.5%)	11 (26.8%)		N/A	
Other/Unknown	46 (19.5%)	9 (22.0%)		N/A	
**English as Primary Language**	193 (83.2%)	38 (95.0%)	0.054	N/A	N/A

**Table 4:** Inaccurate/Missing Contact Information

	No (*n* = 141)	Yes (*n* = 136)	Chi-square *p* value	Pairwise-follow up test *p* value	T-test* p* value
**Male Sex**	81 (58.3%)	81 (59.6%)	0.829	N/A	N/A
**Age**			0.644		N/A
20–39	10 (7.1%)	6 (4.4%)		N/A	
40–59	44 (31.2%)	38 (27.9%)		N/A	
60–79	57 (40.4%)	63 (46.3%)		N/A	
80–99	30 (21.3%)	29 (21.3%)		N/A	
**Ethnicity**			0.519		N/A
African-American, Afro-Carib., Black	30 (21.3%)	36 (26.5%)		N/A	
Asian/Pacific Islander	11 (7.8%)	16 (11.8%)		N/A	
Hispanic	28 (19.9%)	25 (18.4%)		N/A	
White	40 (28.4%)	36 (26.5%)		N/A	
Other/Unknown	32 (22.7%)	23 (16.9%)		N/A	
**English as Primary Language**	123 (89.1%)	108 (80.6%)	**0.049**	N/A	N/A

**Table 5:** Successful Enrollment

	No (*n* = 97)	Yes (*n* = 39)	Chi-square *p* value	Pairwise-follow up test *p* value	T-test* p* value
**Male Sex**	63 (64.9%)	18 (46.2%)	**0.043**	N/A	N/A
**Age**			**0.010**		**0.002**
20–39	6 (6.2%)	0 (0.0%)		0.112	
40–59	33 (34.0%)	5 (12.8%)		**0.013**	
60–79	42 (43.3%)	21 (53.8%)		0.265	
80–99	16 (16.5%)	13 (33.3%)		**0.030**	
**Ethnicity**			0.447		N/A
African-American, Afro-Carib., Black	29 (29.9%)	7 (17.9%)		N/A	
Asian/Pacific Islander	10 (10.3%)	6 (15.4%)		N/A	
Hispanic	17 (17.5%)	8 (20.5%)		N/A	
White	27 (27.8%)	9 (23.1%)		N/A	
Other/Unknown	14 (14.4%)	9 (23.1%)		N/A	
**English as Primary Language**	78 (82.1%)	30 (76.9%)	0.491	N/A	N/A

In investigating patients who were already enrolled in MyChart at the time of discharge (Table 2), statistically significant differences were found based on age (*p* = 0.037) and ethnicity (*p* = 0.049). Gender and English as a primary language did not show significant differences across this point of the care cascade. In regards to age, a pairwise follow-up test revealed that those in the 20–39 age category were statistically more likely to be already enrolled in MyChart (*p* = 0.024), and those 40–59 were statistically less likely to be already enrolled in MyChart (*p* = 0.037). When a pairwise follow-up test was completed for ethnicity, no individual ethnic subpopulation had a statistically significant difference across enrollment groups (*p* > 0.05).

In regards to patients who had missing or inaccurate contact information (Table 3), age was the only demographic category that had statistically significant differences. The average age and standard deviation for the patients not missing contact information was 66.4 years and 15.3 years respectively, and for those with missing contact information it was 60.3 years and 13.5 years respectively. A t-test for age as a continuous variable demonstrated that younger patients had a higher likelihood of having missing contact information (*p* = 0.017). A pairwise follow-up test revealed that only the 40–59 age group were significantly more likely to have missing contact information (*p* = 0.004).

For the next step of the care cascade of successful contact (Table 4), primary language was the only demographic variable found to have statistically significant differences. Patients for whom English is not their primary language were statistically more likely to be successfully contacted compared to their English-speaking peers (*p* = 0.049).

The final step of the care cascade was successful enrollment (Table 5). Men were statistically less likely to be enrolled, despite being successfully contacted, compared to their female counterparts (*p* = 0.043). Statistically significant differences also existed based on age (*p* = 0.010). The average age and standard deviation of the individuals who successfully enrolled was 71.9 years and 11.7 years respectively, contrasted with 63.4 years and 15.3 years respectively for those who were not successfully enrolled. A t-test using age as a continuous variable found that older patients were more likely to successfully enroll compared to younger patients (*p* = 0.002). A pairwise follow-up test found that those in the age group 40–59 were significantly less likely to enroll successfully (*p* = 0.013), and those in the age group 80–99 were more likely to enroll successfully (*p* = 0.030).

### Barriers to enrollment

**Fig. 2:**
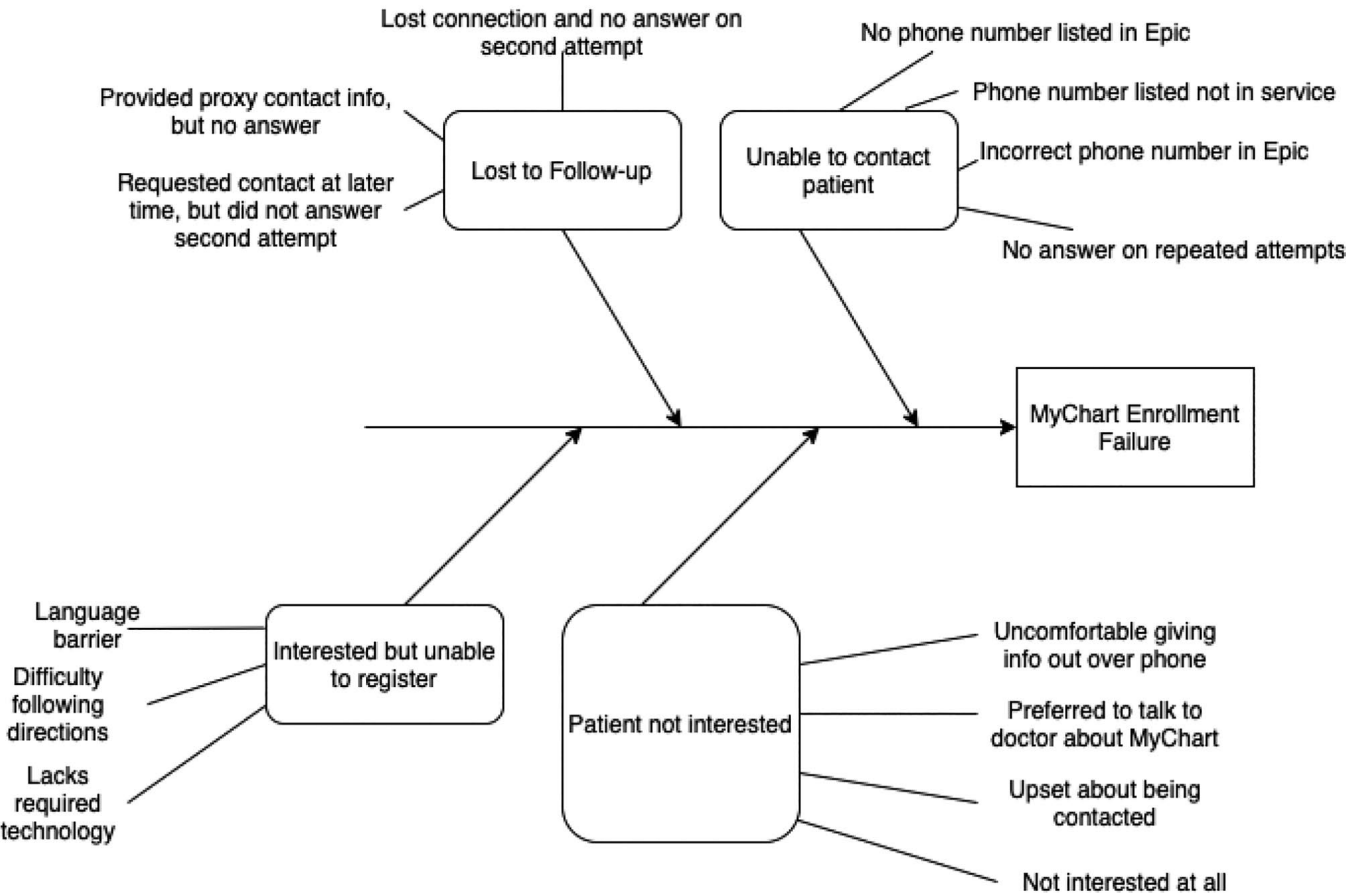
Coding methodology: The authors used a process of comparative analysis to classify barriers to enrollment. Authors looked at individually documented barriers that were transcribed verbatim, and categorized them into various themes. The authors then met to discuss and agree upon a universal classification system that categorized each barrier into a few key groups

**Fig. 3:**
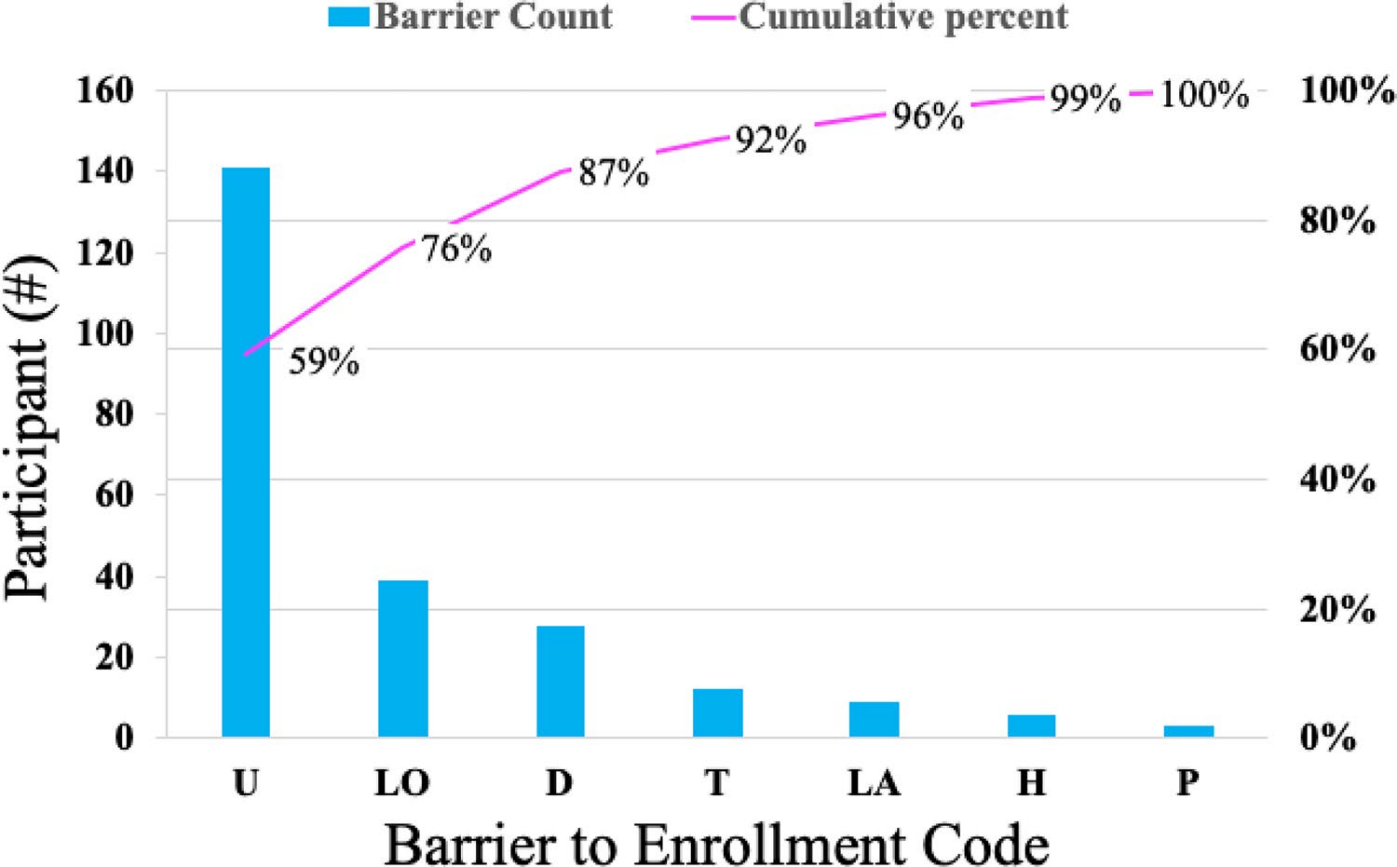
Barriers to MyChart Activation:51% eligible to be enrolled in MyChart were not successfully contacted. An additional 17% became lost to follow-up after initially being contacted, and 9% declined to participate out of lack of interest in remote appointments. Technologic issues, health barriers, language challenges and inability to follow phone directions comprised a smaller percentage of those who did not successfully enroll. U:unable to contact LO:Contacted, loss to follow-up D:Contacted, declined to participate T:Unable to register due to technology challenges LA:Unable to register due to language challenges H:unable to register due to health status P:unable to register due to challenges following phone directions

As previously described, remote outreach failure was a significant barrier to successful patient portal enrollment:over half (51%) of eligible patients could not be contacted. Notably, 15% of eligible patients lacked functional personal phone numbers within their electronic medical record, greatly limiting the outreach capacity of the student volunteers. Of the 136 patients who were successfully contacted, most (71%) of these individuals failed to enroll in MyChart. For these patients, a number of barriers to enrollment were documented and encoded through comparative analysis to classify based on recurring themes (Fig. 2). One such barrier was coded as “lost to follow-up” (Fig. 3, *n* = 39;29% of those contacted):these were patients who were successfully contacted once, but could not complete the enrollment initially and were difficult to reach afterward. It also included patients who asked to be sent activation codes but did not have time to speak to on the phone, and never completed the activation nor answered the phone in subsequent attempts. 28 patients (21% of those contacted) declined to participate due to a lack of interest in conducting a telehealth appointment. Twelve patients (9% of those contacted) stated that technology was a barrier (e.g. not owning a capable device, operator difficulties, etc.). Nine patients (7% of those contacted) were unable to complete enrollment due to the presence of a language barrier (e.g. caller unable to understand the patient on the phone, the patient being prohibitively hard-of-hearing, etc.). Six patients (4% of those contacted) were too ill or tired to discuss MyChart enrollment over the phone, and 3 patients (2% of those contacted) had substantial difficulty in following the enrollment instructions remotely.

## Discussion

The research questions of this study were to (1) assess the viability of remote enrollment compared to in-person enrollment, (2) assess for significant differences along the care cascade in regards to age, gender, ethnicity, and primary language, and (3) to assess the barriers to remotely enrolling patients in a telehealth-capable patient portal. Patients were contacted through either their mobile phones or hospital landlines to reduce SARS-CoV-2 exposure and assist the workflows of the inpatient teams. The results suggest a few key findings.

First, an inability to reach patients was a significant barrier to remote patient portal enrollment. From 2003 to 2010, the number of outpatient medical offices using electronic health records increased from 16 to 52% respectively [33, 34]. Absent data in these systems, in terms of both contact information as well as important medical information, have contributed substantially to poorer health outcomes in the United States [33, 34]. Our data are consistent with these challenges – roughly 15% of our patients did not have a functional personal number in their chart. Our data shows that this was driven particularly by patients in the 40–59 age group – this population was less likely to already be enrolled in MyChart, and more likely to have missing contact information. Contrary to our initial hypothesis, older patients were actually more likely to have contact information and successfully enroll compared to their younger counterparts. The latter may be confounded by the fact that these patients have likely been in the care system for longer, and therefore have had more encounters where missing contact information could be rectified. However, it might also be explained by greater healthcare proxy involvement in this demographic – nearly ¾ of those who successfully enrolled in MyChart had a healthcare proxy do so, and older patients were more likely to successfully enroll compared with younger patients in our sample. It should also be noted that loss to follow-up also contributed substantially to remote enrollment failure. A number of patients in our cohort were successfully contacted but declined assistance enrolling at the time of the call. However, many of these patients failed to complete portal activation and could not be reached on subsequent attempts. These findings suggest that the success of patient portal enrollment is highest when all of the steps are completed at the point of initial contact and that remote enrollment efforts could be improved by confirming patient contact information during each visit. This strategy would be particularly effective if used to target those patients aged 40–59 years, as well as men who were less likely to successfully enroll despite being successfully contacted and would benefit from multiple engagements.

Second, patients’ lack of interest in telehealth as a method of care played a significant role in the failure of eligible patients to complete enrollment. Consistent with previous studies that showed that telemedicine is limited by perceived self-efficacy [21], a number of our patients reported that they were “too old” for telehealth or that the process was “too complicated.” This is likely a byproduct of lack of exposure and knowledge, as well as past healthcare providers having low rates of discussion and education about telehealth initiation. This discomfort around the intersection of health and technology can likely be mitigated by incorporating brief tutorials into in-person primary care appointments to show patients how user-friendly and beneficial telehealth can be. Additionally, as previously discussed, our results showed that three quarters of those who successfully enrolled did so using health care proxies, including spouses, adult children, and paid caregivers. Given that these individuals often provide transportation and/or are present at patients’ in-person health appointments, these can be valuable opportunities to potentially enlist the support of trusted individual that may have higher health and/or technological literacy. Alternatively, healthcare systems can also consider offering free in-person group tutorial sessions for patients to attend to familiarize patients with telehealth technologies employed in their health system, and encouraging patients to bring a family member with them. This approach would normalize lower technological literacy and mitigate perceived threats to patient self-efficacy [21]. For one health system in New York City, its geriatric neurology program has found success in even contacting both individuals in the patient/caregiver dyad simultaneously and automatically when scheduling telehealth appointments, providing an alternative “opt-out” mode of scheduling patient appointments that should also be considered by health systems [35]. Many of our patients in our study did not speak English as a first or primary language, and therefore it will also be important to have interpreter services available at all points of contact with patients and their caregivers. Our study shows that patients for whom English is not a primary language are more likely to be successfully reached to engage in care, and thus this receptive patient population must be appropriately targeted to engage with novel telehealth platforms. These interventions are especially vital considering those most likely to be hesitant with technology, such as the elderly, may be those most vulnerable during an infectious disease surge and, therefore, most likely to benefit from telemedicine.

Third, a number of patients cited technological limitations, such as lacking a device capable of utilizing MyChart or the absence of a wireless internet network at home, as a barrier to completing their post-discharge telehealth visit. While technological constraints are certainly an expected limitation to widespread telemedicine usage, the presence of this barrier within our cohort confirms this to be an important hurdle for telehealth advocates to overcome. Given the increasing role that telemedicine will play in the future of healthcare, leaders in the field ought to devise clever workarounds— such as partnering with insurance companies to loan smart devices to patients or broadening covered telemedical services to include interactions conducted over the phone— if they hope to expand the reach of telehealth capabilities for all patients.

The barriers observed in our study build upon those that have been acknowledged in prior studies on barriers to telehealth enrollment. A number of prior studies have demonstrated that a common barrier to engaging in telehealth is patients’ resistance to change – patients have grown accustomed to face-to-face consultations with their healthcare providers, and do not feel the same connection through a screen [36–38]. In some areas, particularly those more rural, a common barrier to telehealth is that patients simply do not know that telehealth exists [26], and demonstrates the need for healthcare systems to educate the community better in terms of the uses and benefits of telemedicine enrollment, particularly remote enrollment. Our article builds upon these prior studies in two key ways:(1) it investigates barriers in urban populations, as opposed to rural and suburban communities where many prior studies have focused, and (2) it is the first study, to our knowledge, that investigates barriers to remote enrollment, where patients were solicited to join telehealth-delivering portals via phone calls as opposed to in-person. The latter is particularly important in the midst of a global pandemic, where face-to-face solicitations have become increasingly challenging, and potentially unsafe, and therefore offers particular value to health systems developing telehealth infrastructure in this rapidly evolving healthcare landscape.

Finally, the efficacy of our remote enrollment workflow fell short of the success of a recently reported in-person patient portal activation study (14% versus 88% enrollment success) [28]. The populations and settings differed markedly— adult patients admitted to a large metropolitan hospital versus adolescent individuals seen at an outpatient primary care center—and speak to the impact that situation can play in terms of enrollment. Parental influence and involvement in adolescent care likely plays a significant role in higher enrollment success, as well as motivated patients who are already presenting for primary care as opposed to patients with variable medical follow-up presenting with enrollment opportunities after an acute health insult. Additional confounding factors that inhibit the direct comparison of these studies, such as the health statuses of the relative cohorts and the heightened comfortability of adolescent patients with technology, could have had similar effects on enrollment as those differences inherent to remote versus in-person workflows. Indeed, a subset of patients in our cohort reported health status as a barrier to portal activation, and our outcomes also fell short of recent efforts to remotely enroll pediatric patients in a telehealth portal via their parents [29]. However, this deficit in enrollment success taken together with other barriers to remote patient portal activation uncovered here— such as limitations in contacting patients and issues communicating instructions over the phone— suggest that in-person enrollment is superior to remote efforts.

Notable limitations to this study do exist. Our statistical analyses were limited by a sample size of 324, and therefore pairwise follow-up tests investigating individual age categories or ethnicities had less power than investigating significant differences across the entire population. Any attempts to combine subcategories to provide more statistical power (e.g., primary language, which was considered “English” versus “Non-English” for statistical purposes in Table 2) necessitated compiling data in an artificial way that combines heterogenous groups into one. Furthermore, because our task was conducted by volunteers with conflicting schedules, the makeup of our team was subject to change over the course of the study. While a permanent team of individuals would have been preferable, this was not feasible with our volunteer pool nor compatible with the need to contact each patient prior to discharge. Furthermore, due to the time constraints of our volunteers and because patient lists were typically received 48-h prior to discharge, patients received only two remote attempts to enroll in MyChart;increasing the number of outreach attempts per patient may have resulted in increased enrollment success. Finally, volunteers were responsible for transcribing the immediate barrier to patient portal activation in those who failed to enroll. However, it is likely that a subset of patients had multiple barriers to enrollment and that further inquiry could have elicited the complete extent of these barriers in our cohort.

The outcomes of this study set the stage for a few future analyses. Longitudinal analysis of enrolled patients’ telehealth usage, similarly stratified by demographic subgroups, would identify the success of these efforts beyond initial patient portal activation. Additionally, following the conclusion of our student volunteer efforts, the MSBI Department of Medicine transitioned to in-person MyChart enrollment during patients’ pre-discharge workflow. A comparison of our remote versus in-person data would further illustrate the superior method of telehealth portal enrollment and represents a future goal of this work.

Within our cohort of over 300 patients discharged from MSBI, 85% of individuals were not yet enrolled in the hospital’s affiliated patient portal application and were expected to complete a telehealth follow-up visit within the week. Despite the efforts of our volunteers, just 14% of eligible volunteers could be enrolled remotely. Those unable to be enrolled at the time were recontacted starting in May 2020 when clinics reopened for in-person appointments. However, these gains still represent an increase of 83% in MyChart enrollment compared to those who were already enrolled, indicating remote patient portal activation represents a feasible route for bolstering telehealth capacity if in-person methods are not available. Remote enrollment efforts are undoubtedly improved by assuring that patients’ contact information is correct during each encounter and enlisting the assistance of healthcare proxies.

## Conclusions

For health system leaders aiming to improve their telemedical infrastructure, we recommend incorporating patient portal enrollment into existing in-person workflows, such as inpatient nursing teams’ pre-discharge checklists or outpatient administrative assistants’ checkout protocols, and ensuring during these encounters that patient contact information is both documented and correct. Telehealth’s overall reach could be further broadened by considering specific barriers to access related to individual demographic groups (including technological, linguistic, and educational barriers outlined in this article), hosting in-person tutorials/seminars on telehealth for patients and inviting family to join the patient, partnering with insurance companies to overcome patients’ coverage limitations, and trusted healthcare providers explaining the importance of telehealth to patients in this evolving healthcare environment. Our results suggest that male patients, and patients aged 40–59 are particularly to benefit from these interventions. This study’s conclusions are limited by a compressed time frame over which contact was attempted for individual patients due to clinics reopening for in-person consultations, simplification of barriers to enrollment to a single reason, and changing personnel involved in the research team as availabilities within the early pandemic fluctuated. Despite this, our data provide important lessons for the future management of telehealth opportunities during the COVID-19 pandemic as well as future infectious diseases crises, and outline targets to improve enrollment in telehealth services so that our hospital systems can rise to meet the needs of our rapidly evolving healthcare landscape.

## Data Availability

All data is available upon request.

## References

[1] American Hospital Association. Fact Sheet:Telehealth. 2019. Accessed on 1 August 2021 at https://www.aha.org/system/files/2019-02/fact-sheet-telehealth-2-4-19.pdf.

[2] Ray KN, Chari AV, Endberg J, et al. Opportunity costs of ambulatory care in the United States. Am J Manage Care. 2015;21(8). Accessed on 1 August 1, 2021 at https://www.ajmc.com/view/opportunity-costs-of-ambulatory-medical-care-in-the-united-states. PMC8085714 26295356

[3] Ray KN, Chari AV, Endberg J, et al. Disparities in time sent seeking medical care in the United States. JAMA Intern Med. 2015;175(12):1983–1986. Accessed on 1 August 2021 at https://www.ncbi.nlm.nih.gov/pmc/articles/PMC5055855/. 10.1001/jamainternmed.2015.4468 PMC5055855 26437386

[4] Ornstein KA, Leff B, Covinsky KE, et al. Epidemiology of the homebound population in the United States. JAMA Intern Med. 2015;175(7):1180–1186. Accessed on 1 August 2021 at https://www.ncbi.nlm.nih.gov/pmc/articles/PMC4749137/. 10.1001/jamainternmed.2015.1849 PMC4749137 26010119

[5] Coffman M, Moore M, Jetty A, et al. Who is using telehealth in primary care? Safety net clinics and health maintenance organizations (HMOs). J Am Board Fam Med. 2016;29(4):432–433. Accessed on 1 August 2021 at https://www.jabfm.org/content/29/4/432.long. 10.3122/jabfm.2016.04.150375 27390373

[6] Farahani N, Pantanowitz L. Overview of telepathology. Surg Pathol Clin. 2015;8(2):223–231. Accessed on 1 August 2021 at https://linkinghub.elsevier.com/retrieve/pii/S1875-9181(15)00036-7 10.1016/j.path.2015.02.018 26065796

[7] Sirintrapun SJ, Lopez AM. Telemedicine in Cancer Care. Am Soc Clin Oncol Educ Book. 2018;38:540–545. Accessed on 1 August 2021 at 10.1200/EDBK_200141. 10.1200/EDBK_200141 30231354

[8] Norman S. The use of telemedicine in psychiatry. J Psychiatr Mental Health Nursing. 2006;13(6):771–777. Accessed on 1 August 2021 at https://onlinelibrary.wiley.com/10.1111/j.1365-2850.2006.01033.x. 10.1111/j.1365-2850.2006.01033.x 17087682

[9] Buchheit BM, Wheelock H, Lee A, et al. Low-barrier buprenorphine during the COVID-19 pandemic:A rapid transition to on-demand telemedicine with wide-ranging effects. J Subst Abuse Treatment. 2021;131:108444. Accessed on 1 August 2021 at https://www.ncbi.nlm.nih.gov/pmc/articles/PMC8081577/pdf/main.pdf. 10.1016/j.jsat.2021.108444 PMC8081577 34098299

[10] Miller MJ, Pak SS, Keller DR, et al. Evaluation of pragmatic telehealth physical therapy implementation during the COVID-19 pandemic. Phys Therapy. 2021;101(1):pzaa193. Accessed on 1 August 2021 at https://www.ncbi.nlm.nih.gov/pmc/articles/PMC7665714/. 10.1093/ptj/pzaa193 PMC7665714 33284318

[11] Tresenriter M, Holdaway J, Killeen J, et al. The implementation of an emergency medicine telehealth system during a pandemic. J Emergency Med. 2021;60(4):548–553. Accessed on 1 August 2021 at https://www.ncbi.nlm.nih.gov/pmc/articles/PMC7789960/. 10.1016/j.jemermed.2020.11.026 PMC7789960 33423835

[12] Weber AM, Dua A, Cheng K, et al. An outpatient telehealth elective for displaced clinical learners during the COVID-19 pandemic. BMC Med Educ. 2021;21(174). Accessed on 1 August 2021 at https://bmcmededuc.biomedcentral.com/track/pdf/10.1186/s12909-021-02604-z.pdf. 10.1186/s12909-021-02604-z PMC7980791 33743676

[13] Coccia M. Sources of technological innovation:Radical and incremental innovation problem-driven to support competitive advantage of firms. Technol Anal Strateg Manag. 2016;29(9). Accessed on 1 August 2021 at https://www.researchgate.net/publication/311993766_Sources_of_technological_innovation_Radical_and_incremental_innovation_problem-driven_to_support_competitive_advantage_of_firms.

[14] Coccia M. Sources of disruptive technologies for industrial change. L’industria 2017;XXXVIII(1):97–112. Accessed on 1 August 2021 at https://www.researchgate.net/publication/319098829_Sources_of_disruptive_technologies_for_industrial_change.

[15] Coccia M. Technological parasitism. J Econ Social Thought. 2019;6(3):173–209. Accessed on 1 August 2021 at https://www.proquest.com/openview/1d72892cb9bcfa5db38dc316fbc7cad9/1?pq-origsite=gscholar&cbl=2041976.

[16] Coccia M, Watts J. A theory of the evolution of technology:*Technological parasitism* and the implications for innovation management. J Eng Technol Manag. 2020;55:101552. Accessed on 1 August 2021 at https://www.sciencedirect.com/science/article/abs/pii/S0923474818304211.

[17] Gajarawala SN, Pelkowski JN. Telehealth benefits and barriers. J Nurse Pract. 2021;17(2):218–221. Accessed on 1 August 2021 at https://www.ncbi.nlm.nih.gov/pmc/articles/PMC7577680/pdf/main.pdf. 10.1016/j.nurpra.2020.09.013 PMC7577680 33106751

[18] Weigel G, Ramaswamy A, Sobel L, et al. Opportunities and barriers for telemedicine in the US during the COVID-19 emergency and beyond. Kaiser Family Foundation. 2020. Accessed on 1 August 2021 at https://www.kff.org/womens-health-policy/issue-brief/opportunities-and-barriers-for-telemedicine-in-the-u-s-during-the-covid-19-emergency-and-beyond/.

[19] Lin CCC, Dievler A, Robbins C, et al. Health Affairs. 2018;37(12):1967–1974. Accessed on 1 August 2021 at https://pubmed.ncbi.nlm.nih.gov/30633683/. 10.1377/hlthaff.2018.05125 30633683

[20] Weinstein RS, Lopez AM, Joseph BA, et al. Telemedicine, telehealth, and mobile health applications that work:Opportunities and barriers. Am J Med. 2014;127(3):183–187. Accessed on 1 August 2021 at https://www.amjmed.com/article/S0002-9343(13)00919-4/fulltext. 10.1016/j.amjmed.2013.09.032 24384059

[21] Sanders C, Rogers A, Bowen R (2012). Exploring barriers to participation and adoption of telehealth and telecare within the Whole System Demonstrator trial:a qualitative study. BMC Health Serv Res.

[22] Donelan K, Barreto EA, Sossong S, et al. Patient and clinician experiences with telehealth for patient follow-up care. Am J Manag Care. 2019;25(1):40–44. Accesed on 1 August 2021 at https://www.ajmc.com/view/patient-and-clinician-experiences-with-telehealth-for-patient-followup-care. 30667610

[23] Rashedi J, Mahdavi PB, Asgharzadeh V, et al. Risk Factors for COVID-19. Le Infezioni in Medicina. 2020;28(4):469–474. Accessed on 1 August 2021 at https://pubmed.ncbi.nlm.nih.gov/33257620/. 33257620

[24] Koonin LM, Hoots B, Tsang CA, et al. Trends in the use of telehealth during the Emergence of the COVID-19 pandemic – United States, January-March 2020. MMWR Morb Mortal Wkly Rep. 2020;69:1595–1599. Accessed on 1 August 2021 at https://www.cdc.gov/mmwr/volumes/69/wr/mm6943a3.htm. 10.15585/mmwr.mm6943a3 PMC7641006 33119561

[25] Demeke HB, Pao LZ, Clark H, et al. Telehealth practice among health centers during the COVID-19 pandemic – United States, July 11–17, 2020. MMWR Morb Mortal Wkly Rep. 2020;69:1902–1905. Acessed on 1 August 2021 at https://www.cdc.gov/mmwr/volumes/69/wr/mm6950a4.htm. 10.15585/mmwr.mm6950a4 PMC7745961 33332297

[26] Rutledge CM, Haney T, Bordelon M, et al. Telehealth:Preparing advanced practice nurses to address healthcare needs in rural and underserved populations. Int J Nursing Educ Scholarsh. 2014;11. Accessed on 1 August 2021 at https://www.degruyter.com/document/10.1515/ijnes-2013-0061/html. 10.1515/ijnes-2013-0061 24423469

[27] Ronis SD, McConnochie KM, Wang H, et al. Urban telemedicine enables equity in access to acute illness care. Telehealth J E-health. 2017;23(2):105–112. Accessed on 1 August 2021 at https://pubmed.ncbi.nlm.nih.gov/27383822/. 10.1089/tmj.2016.0098 27383822

[28] Ramsey A, Lanzo E, Huston-Paterson H, et al. Increasing patient portal usage:Preliminary outcomes from the MyChart Genius Project. J Adolesc Health. 2018;62(1):29–35. Accessed on 1 August 2021 at https://www.ncbi.nlm.nih.gov/pmc/articles/PMC5963535/. 10.1016/j.jadohealth.2017.08.029 PMC5963535 29169768

[29] Patel PD, Cobb J, Wright D, et al. Rapid development of telehealth capabilities within pediatric patient portal infrastructure for COVID-19 care:Barriers, solutions, results. Published online ahead of print. J Am Med Inform Assoc. 2020;ocaa065. Accessed on 1 August 2021 at https://www.ncbi.nlm.nih.gov/pmc/articles/PMC7188108/. 10.1093/jamia/ocaa065 PMC7188108 32302395

[30] Xu S, Li Y. Beware of the second wave of COVID-19. Lancet. 2020;395(10233):1321–1322. Accessed on 1 August 21 at https://www.ncbi.nlm.nih.gov/pmc/articles/PMC7194658/. 10.1016/S0140-6736(20)30845-X PMC7194658 32277876

[31] Coccia M. An index to quantify environmental risk of exposure to future epidemics of the COVID-19 and similar viral agents:Theory and practice. Environ Res. 2020;191:110155. Accessed on 1 August 2021 at https://www.ncbi.nlm.nih.gov/pmc/articles/PMC7834384/. 10.1016/j.envres.2020.110155 PMC7834384 32871151

[32] Raman R, Patel KJ, Ranjan K. COVID-19:Unmasking emerging SARS-CoV-2 variants vaccines, and therapeutic strategies. Biomolecules. 2021;11(7):993. Accessed on 1 August 2021 at https://www.ncbi.nlm.nih.gov/pmc/articles/PMC8301790/. 10.3390/biom11070993 PMC8301790 34356617

[33] Wells BJ, Chagin KM, Nowacki AS, et al. Strategies for handling missing data in electronic health record derived data. EGEMS. 2013;1(3):1035. Accessed on 1 August 2021 at https://www.ncbi.nlm.nih.gov/pmc/articles/PMC4371484/. 10.13063/2327-9214.1035 PMC4371484 25848578

[34] Kokkonen EWJ, Davis SA, Lin HC, et al. Use of electronic medical records differs by specialty and office settings. J Am Med Info Assoc. 2013;20(e1):e33–38. Accessed on 1 August 2021 at https://pubmed.ncbi.nlm.nih.gov/23538721/. 10.1136/amiajnl-2012-001609 PMC3715335 23538721

[35] Weiss EF, Malik R, Santos T, et al. Telehealth for the cognitively impaired older adult and their caregivers:Lessons from a coordinated approach. Neurodegener Dis Manag. 2020;11(1):83–89. Accessed on 7 October 2021 at https://www.futuremedicine.com/10.2217/nmt-2020-0041. 10.2217/nmt-2020-0041 PMC7659596 33172352

[36] Young LB, Chan PS, Cram P. Staff acceptance of tele-ICU coverage. Chest. 2011;139(2):279–288. Accessed on 1 August 2021 at https://www.ncbi.nlm.nih.gov/pmc/articles/PMC3032369/. 10.1378/chest.10-1795 PMC3032369 21051386

[37] LeRouge C, Garfield MJ. Crossing the telemedicine chasm:Have the US barriers to widespread adoption of telemedicine been significant reduced? Int J Environ Res Pub Health. 2013;10(12):6472–6484. Accessed on 1 August 2021 at https://www.ncbi.nlm.nih.gov/pmc/articles/PMC3881125/. 10.3390/ijerph10126472 PMC3881125 24287864

[38] Kruse CS, Karem P, Shifflett K, et al. Evaluating barriers to adopting telemedicine worldwide:A systematic review. J Telemed Telecare. 2018;24(1):4–12. Accessed on 1 August 2021 at https://www.ncbi.nlm.nih.gov/pmc/articles/PMC5768250/. 10.1177/1357633X16674087 PMC5768250 29320966

